# The Study of Usefulness of a Set of Fractal Parameters to Build Classes of Disease Units Based on Images of Pigmented Skin Lesions

**DOI:** 10.3390/diagnostics11101773

**Published:** 2021-09-26

**Authors:** Monika Styła, Tomasz Giżewski

**Affiliations:** 1 Chair and Department of Biophysics, Medical University of Lublin, 20-090 Lublin, Poland; 2 Department of Electrical Engineering and Electrotechnologies, Faculty of Electrical Engineering and Computer Science, Lublin University of Technology, 20-618 Lublin, Poland; t.gizewski@pollub.pl

**Keywords:** dermatology, classifications, pigmented skin lesions, fractal analysis

## Abstract

Dermatoscopic images are also increasingly used to train artificial neural networks for the future to provide fully automatic diagnostic systems capable of determining the type of pigmented skin lesion. Therefore, fractal analysis was used in this study to measure the irregularity of pigmented skin lesion surfaces. This paper presents selected results from individual stages of preliminary processing of the dermatoscopic image on pigmented skin lesion, in which fractal analysis was used and referred to the effectiveness of classification by fuzzy or statistical methods. Classification of the first unsupervised stage was performed using the method of analysis of scatter graphs and the fuzzy method using the Kohonen network. The results of the Kohonen network learning process with an input vector consisting of eight elements prove that neuronal activation requires a larger learning set with greater differentiation. For the same training conditions, the final results are at a higher level and can be classified as weaker. Statistics of factor analysis were proposed, allowing for the reduction in variables, and the directions of further studies were indicated.

## 1. Introduction

Technological development over the last few decades has contributed to the introduction of new tools aimed at monitoring and proper diagnosis of skin diseases. Dermatoscopy [1,2,3] enables a more accurate assessment of pigmented lesions compared to the examination carried out with the naked eye, which increases the effectiveness of early detection of precancerous lesions. It may also be helpful in the diagnosis of other dermatological features. However, the main purpose of the dermatoscopic examination is to confirm or rule out the diagnosis of malignant melanoma, and dermatoscopic images can also be analyzed using computer applications.

In the interpretation of medical data, regardless of the type of imaging, there are problems related to the scatter of parameters resulting from the apparatus resolution, but also subjective evaluation of results. For example, in tests of eye fundus and diabetic retinopathy, a measure of capillary blood vessel coverage is important in the case of dermatological diseases—the assessment of color, degree of discoloration, structure or gradient limit [4,5,6]. All of these elements constitute the problem of the qualification process (based on the physician’s experience) to make a proper diagnosis.

The main element of the clinical image, which is the basis for the diagnosis of dermatological diseases, are skin lesions [7,8]. Description of the lesions takes into account both morphological features of the lesions (shape, area, restriction of location, form, number, color, size, evolution, scale off) and subjective symptoms (soreness, burning skin, itching) [1,9].

All these parameters allow identifying symptoms of a malignant lesion based on the observation of a set of characteristics using dermatoscopic images. Unfortunately, in some cases, it could be a difficult task to interpret these properties visually, and therefore, to make the right diagnosis. For many years, scientists have been looking for new techniques [10,11,12,13] combined to improve the accuracy of the classifications. 

One of the methods of melanoma classification is the automatic extraction of characteristics that take into account the rule ABCD [14,15] (asymmetry, border, color, diameter). In contrast to conventional methods of surface evaluation, the fact that although it contains a large number of heterogeneities, the parameters characterizing it depends on the adopted scale. A logical solution is to use the same size at different scales. In this context, the concept of fractals (lat. fractus) [16,17,18,19,20] is used to describe the roughness of a surface, which describes objects with irregular shapes.

In our analysis, we focused on the ABCD scale containing the most important features because, in the study by the authors of [21], it is the comparison of sensitivity, specificity and diagnostic accuracy of the ABCD rule and the seven-point checklist for the diagnosis of melanocytic lesions on the skin. In the study, the ABCD rule of dermatoscopy showed the highest sensitivity for the diagnosis of melanoma. Extending a study on the seven-point checklist rule would only provide a comparative value between the two scales (ABCD vs. seven-point checklist).

Fractal parameters are also widely used in other medical fields. Fractal analysis can be useful in cardiology. The studies by Lawrence et al. [22] show that fractals can help measure the changes in the microstructure of blood clots. Beckers et al. [23] proved that fractal dimension analysis could help observe physiological changes taking place in the myocardium.

The fractal analysis enables the diagnosis of cancerous lesions in oncology. Zheng et al. [24] extracted a lot of parenchymal texture features from the entire breast region that might improve breast cancer risk assessment. Daye et al. [25] investigated differences in texture features between pre- and post-menopausal women, which may be attributed to an increased association to breast cancer risk.

Studies [26,27] proved that fractal analysis could be considered a pre-test to measure bone density, normally used in the diagnosis of osteoporosis. Lacunarity and fractal dimension combined with X-rays can also be useful in dentistry to evaluate endodontic treatment [28].

Besides the examples mentioned above, fractal analysis can be using wide industrial applications, i.e., in civil engineering [29,30], material science [31,32], geology [33,34], chemical sciences [35,36] and automotive [37,38].

The fractal analysis method in support of medical diagnosis is a promising tool in the assessment of image parameters, regardless of the adopted scale. Therefore, such studies are still justified and are carried out in many scientific research centers [8,39].

## 2. Materials and Methods

The initial assumptions concerning the examination of pigmented skin lesions by means of fractal analysis of images carried out with a videodermatoscopy included the creation of a computer program for automatic pre-processing and determination of values: fractal dimension [40,41,42], lacunarity [43,44,45] and surface for red, green, blue and averaged to grayscale components. The above-mentioned functions were aimed at distinguishing clear features of dermatological lesions enabling the construction of a machine learning algorithm. Classification of the image in groups of similarities required the unambiguous separation of simple features in numbers allowing to build an input vector with the greatest possible degree of freedom.

**Figure 1:**
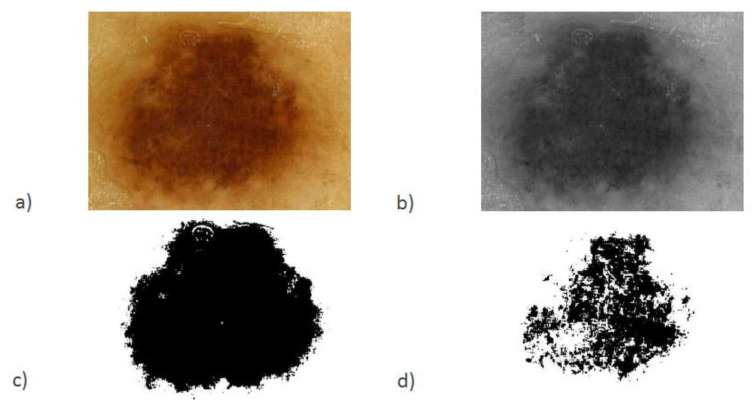
Pigmented skin lesion image: (**a**) an example, (**b**) converted to grayscale mode, (**c**) binarization based on the threshold determined for the minimum value of the histogram, (**d**) binarization based on the threshold determined for the value of the first maximum histogram.

The basic classification algorithm determined the rules of processing photos of pigmented skin lesions. In the conducted studies, an algorithm of image division into three components and their binarization [46,47,48] with additional analysis of the binarization grayscale image was assumed. Figure 1 presents one of the hundred images we analyzed in the study.

A typical image from videodermatoscopy is a single-layer 96 dpi image of size 1920 × 1080 with a 24-bit representation of red, green and blue components (R—red, G—green, B—blue). Magnification in a standard test is *m* = 20.

The program is equipped with tools to apply filters in order to detect edges or contrast specific features of pigmented lesions. It performs an assessment of the fractal dimension by box-counting, lacunarity by mass radius and estimation of the area of significant surface for individual components.

### 2.1. Determination of Fractal Parameters

The basic program function after pre-processing is to evaluate the fractal dimension, lacunarity and the surface area of the pigmented lesion in the binarization image. By taking into account the self-similarity features, the fractal dimension was calculated.

In the case of fractal analyses of images without clear self-similarity features, the methods of searching for patterns should be used with the determination of their occurrence probability. Among the known methods (the reticular cell counting method, Keller approach, differential box-counting method), the method of counting surfaces of equal color in the whole subarea was chosen.

An algorithm was implemented to determine the size of a single square, which is the total size divisor of the raster area in the analyzed image. In the procedure, an algorithm for counting squares of equal color was implemented. In the case of a binary image, only two colors were available: black and white. In the saved algorithm, iteration was carried out on all elements of the array. In subsequent iterations with respect to the *m* variable, the size of the element was determined in each step, and then, by dividing the active image into equal parts of the defined array size, each part of the image was compared with the pattern with total white or black color. The procedure counted all elements of the same color.

For each determined value of the window size *r*, the relation ln(*N*) to ln(*r*^−1^) was plotted, where *N* was the number of similar squares. The resulting discrete distribution of points was proximized by a line. Its directional coefficient was determined by the method of the smallest squares, and it determined the fractal dimension of a surface object. Its value was within limits (2:3). Depending on the iteration with respect to the size of the smallest to the largest square, the directional coefficient had a negative value; otherwise, it was positive. The absolute value of the directional factor was understood as a fractal dimension. The fractal dimension, for the purpose of dermatological image analysis, was calculated for all color components separately and for the grayscale. The procedure was carried out following the binarization of each of them.

Lacunarity is defined as the dimensionless coefficient of first and second-order mass distribution. Mass, as understood by the image, is defined as the grey level of a single pixel. The program has applied a lacunarity test for a binary image for which the pixel mass may be 0 or 255.

In practice, the algorithm must take into account the probability of an element of size *r* × *r* with specified acceptable mass distribution in the whole image. One of the functions used counts the mass of a single box, taking the weight of a single white point as 0 and the weight of a black point as 1. The lacunarity is calculated by the implemented algorithm.

The last parameter calculated in the program is the surface area of binarization images. This feature is determined from the number of blackened points and is a good approximation of the surface area in the color change. The total area is taken as the number for the threshold of the minimum histogram value and the sum of multiple color components.

### 2.2. Input Data Vector

The image pre-processing [49,50,51] and its fractal analysis allowed to determine its basic parameters, such as the fractal dimensions for individual color components binarized with respect to the minimum histogram in the grayscale (e.g., fractal dimension for the red component *D_r_−*01) or the fractal dimension of image averaged to the grayscale *D_s_−*01. The lacunarity for individual color components binarized with respect to the greyscale minimum (e.g., lacunarity for the red component *L_r_−*01) and the lacunarity of image averaged to the greyscale *L_s_−*01 were also determined. In addition, the surface area for components of individual colors binarized with respect to the minimum histogram in the grayscale (e.g., surface area for the red component *A_r_−*01) and the surface area of image averaged to the grayscale, *A_s_−*01, were calculated. Moreover, the same parameters (fractal dimension, lacunarity and surface area) binarized with respect to the first maximum histogram in the grayscale (e.g., fractal dimension for the red component *D_r_−*02, lacunarity for the red component *L_r_−*02, surface area for the red component *A_r_−*02) were determined analogously. 

**Figure 2:**
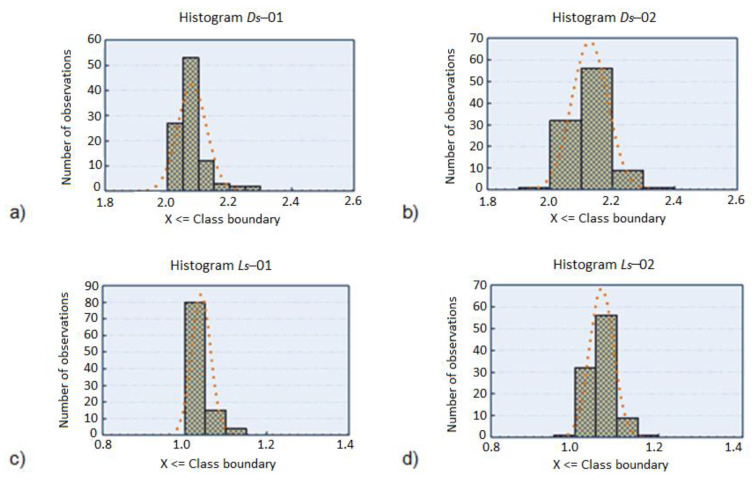
Histograms for variables: (**a**) *D_r_−*01, (**b**) *D_r_−*02 with class 0.2 limits from 1.8 to 2.6, mm^2^, (**c**) *L_s_−*01, (**d**) *L_s_−*02 with class 0.2 limits from 0.8 to 1.4 mm^2^.

In the first stage of preliminary classification, the ranges of variables and their frequency of occurrence were determined, and the selected results were presented in the form of histograms for each variable separately Figure 2.

On the basis of histograms of variable *D_s_*, which is a fractal dimension calculated for an image averaged to grayscale, binarized for two threshold values, a preliminary classification formed by dividing the range of variation into 6 categories (binarization based on histogram minimum) can be observed. There is also a visible decrease in the number of sets with similar elements, which is caused by lowering the binarization threshold to the first maximum. In addition, this introduces the possibility of incorrect classification due to the fractal dimension below two, which occurs in one case. 

Based on the histogram of variable *L_s_*, denoting the lacunarity value calculated for the image averaged to the grayscale, binarized with respect to the histogram minimum, it is possible to notice the occurrence of an initial number of classes formed by dividing the range of variability into 3 categories. The highest number of cases is assigned to the class, the range of which is formed by a lacunarity value from 1 to 1.05. Lowering the binary threshold to the first maximum increased the number of sets with similar elements and caused the occurrence of classes with values from the ranges below 1 and above 1.15 (one element in each of them).

**Figure 3:**
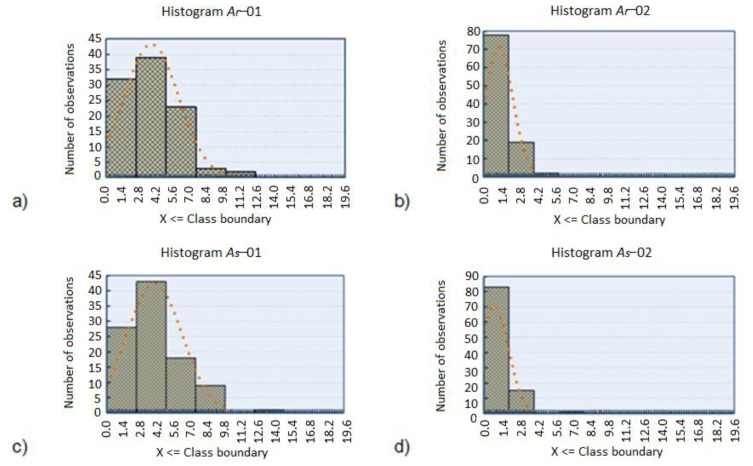
Histograms for variables: (**a**) *A_r_−*01, (**b**) *A_r_−*02 with class 0.2 limits from 0 to 20 mm^2^, (**c**) *A_s_−*01 and (**d**) *A_s_−*02 with class 0.2 limits from 0 to 20 mm^2^.

In the following Figure 3, histograms for surface fields of image categories for two binarization thresholds were compiled. Due to interesting parameters in the classification of objects, they were omitted as the quantities characterizing the classes. They are used in the statistical analysis only as independent variables.

The analysis of sample size in the set of images indicates that the largest proportion of pigmented lesions occurs in red areas in the range below 7 mm^2^. This value is determined on the basis of the minimum histogram, which was taken as a starting point for determining the area of pigmented lesion. Similar results are obtained for histograms for other variables (*A_g_*, *A_b_*), not including in this paper.

On the basis of analysis of the above histograms, an input data vector was developed, the ordered form of which was arranged in the following way: *D_r_−*01—actual value with 3 decimal places; *L_r_−*02—actual value with 3 decimal places; *A_s_−*02—actual value with 3 decimal places, etc.

## 3. Results

The search for sets of similar objects was carried out by means of a preliminary analysis of correlations for individual variables of the input vector and the Kohonen unsupervised neural classifier.

The set of input features, which consists of fractal dimensions for individual color components and an averaged grayscale image for two threshold levels, constitutes the variables dependent on the preliminary analysis carried out by statistical methods.

### 3.1. Preliminary Classification by Unsupervised Statistical Methods

The first stage of statistical research concerned the analysis of correlation of fractal dimension and lacunarity variables for all components of color and averaged grayscale image, respectively. The results of the compilation of the Pearson correlation coefficient show a value at the significance level below 0.05 was considered a significant result, and the correlation is strong. The remaining correlations in the images of binarized red, green and blue levels are weak. For an average grayscale image, the correlations depend on the leading color level.

**Figure 4:**
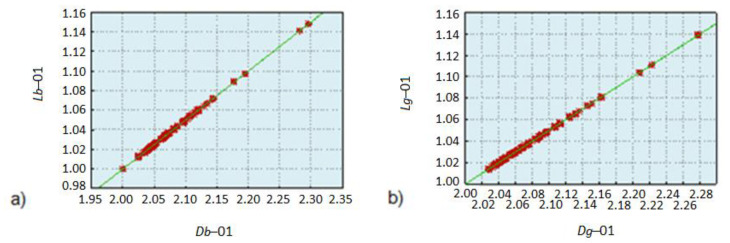
Correlation and scatter graph of the variable fractal dimension in relation to lacunarity for (**a**) blue components and (**b**) fractal dimension calculated for an image averaged to grayscale.

Figure 4 illustrates the scatter and correlation graphs for fractal dimension and lacunarity variables of blue components and for the grayscale. The coordinates of points are arranged along a single line, which is also reflected in the Pearson correlation coefficient approximately equal to 1.

From the above analyses, it follows that the group of variables, lacunarity or fractal dimension, may be omitted from the initial classification. The linear correlation does not bring significant identification value but increases the dimension of the teaching vector.

The results of studies on the relation between the area of color change surface and the fractal dimensions by means of correlation analysis and especially subjective interpretation of the scatter graphs show the dispersion of the collection elements. The qualitative graph indicates the possibility of creating classes with the largest number of samples. These classes can be subjected to medical analysis to assign the diagnosis. 

The change of binarization threshold to the first maximum changes the scatter distribution of elements on the plane of fractal dimension in relation to the area of color change surface. It was noticed that the fractal dimension for lowered binarization threshold changes in negative correlation. This proves the claim that the binarization threshold should be selected in such a way as to obtain the maximum scatter of values. This allows more classes for disease units to be created.

**Figure 5:**
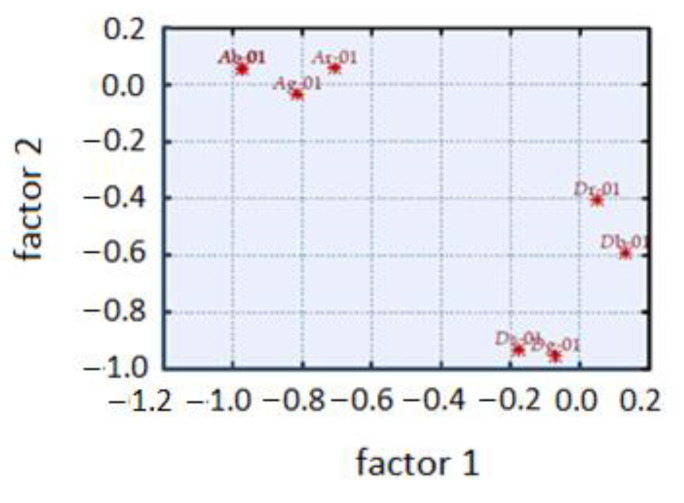
Factor analysis for the variables of the fractal dimension of RGB components and averaged grayscale.

The result of the factor analysis presented in Figure 5 indicates the possibility of reducing the input variables in the classification and, consequently, in the identification of objects. This will significantly affect the form of the set of classes and simplify the analysis of multidimensional objects. The figure shows that out of 100 images analyzed by the program, three disease units of skin lesions were created. It coincides with the information obtained from the dermatologists who provided us the images for our analysis.

Statistical analyses carried out to classify pigmented skin lesions should be considered as a preliminary knowledge base. The results and experience gained during the research on fractal analysis in dermatological diagnostics indicate the need for more complex statistical research. However, they go beyond the scope of this paper.

### 3.2. Neural Classifier—Kohonen Network

The Kohonen network is used in general cybernetics as a neural classifier. Due to the preliminary assumptions made in this paper, i.e., the study of the usefulness of a set of fractal parameters to build classes of disease units based on images of pigmented skin lesions, the Kohonen artificial neural network algorithm is the basic choice of tool for the analysis.

In general, the algorithm is widely recognized in neurobiology, where many structures, e.g., in the brain, have linear and plane topologies, described by a one-dimensional or two-dimensional space. A simple experience, such as color perception, excites three different types of receptors. The human eye perceives various signals about the structure, position and texture of an object. Signals from individual receptors are transferred to the brain structures, which are subject to interpretation. The above example shows that the input parameters are multidimensional and always ordered.

**Figure 6:**
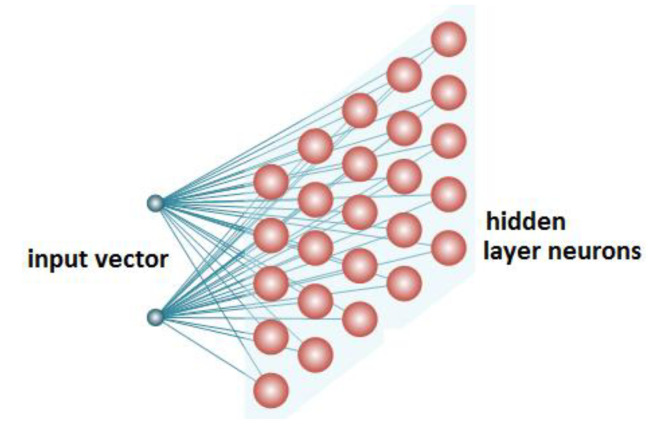
A simplified diagram of a Kohonen neural network.

The system, which is supposed to realize the functioning of a self-organizing network, should consist of two basic elements: a matrix of neurons that are stimulated by input signals, a mechanism that defines the stage of similarity of every neuron with an input signal and output hidden layers vector (Figure 6).

All of the input neurons are connected to all of the output neurons, and these connections have weights, or strengths, associated with them. When the network is fully trained, data that are similar should be close together on the output vector, whereas data that are vastly different will be far apart.

The study of classes of disease units based on images of pigmented skin lesions was carried out by analyzing the convergence of learning error and validation functions. A set of fractal parameters and the surface area of the binarized image of each component were used as input data vectors.

In the first stage, it was proposed to classify without supervision the artificial neural network algorithm of the Kohonen model, performed for an input vector, consisting of variables *D_r_, D_g_, D_b_, D_s_, A_r_, A_g_, A_b_* and *A_s_*, for values calculated on the basis of binarized images with respect to a minimum color histogram. The structure of the 8 × 8 model was proposed.

**Figure 7:**
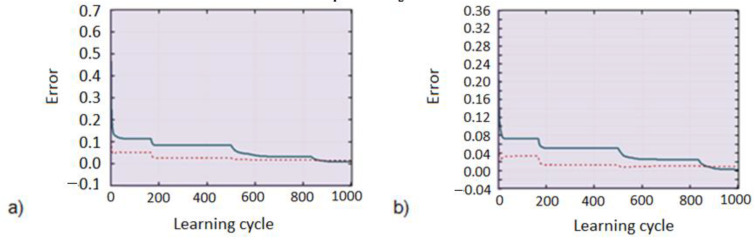
Kohonen network learning and testing error graph in 8 × 8 structure, for the eight-element input vector: (**a**) solid blue line—learning error, dotted red line—testing error, (**b**) solid blue line—learning error, dotted red line—testing error.

The form of the input vector, consisting of eight variables and the structure of the artificial neural network model, created a classifier with 64 active class indicators. The learning graph indicates that the classifier minimizes the learning error to less than 1% after 800 learning cycles (cf. Figure 7a). Table 1 compiles sample test data with the coordinates of the winning neuron.

Part b in the figure below shows a graph of the learning error and the Kohonen network test with a reduced input vector to five variables: fractal dimensions and one area of the red component. A slower rate of minimizing the learning error and lower differentiation of neuronal activation were observed. The reason is that less information is introduced into the structure, and therefore it is more difficult to determine the diversity of classes. The solution to the problem may be to increase the learning set or reduce the number of classes by reducing the number of neurons in the structure.

The Kohonen neural network in each winning neuron stores information about the object class. The graph below illustrates the levels of neuronal activity. On their basis, a conclusion was formulated on the reduction in the number of input vector variables and the number of classes (network structure).

**Figure 8:**
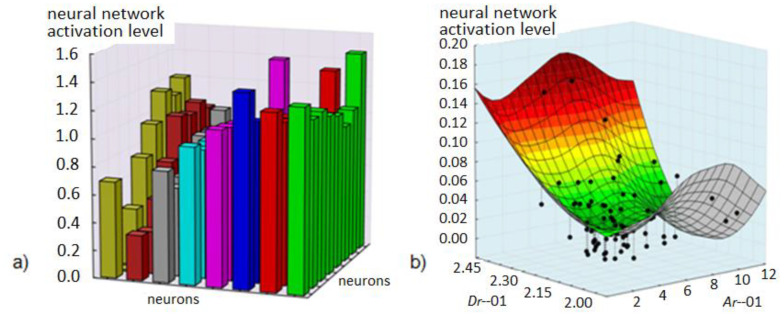
Kohonen neural network activation levels: (**a**) in 8 × 8 structure, (**b**) in 8 × 8 structure for continuous pair of variables *D_r_*−01 and *A_r_*−01.

Figure 8 presents continuous graphs of neuronal activation as a function of fractal dimensions *Dr*−01 and change area fields in the red component image. Three classes of object similarity can be inferred after preliminary analysis. In the component, four areas with potential classes can be distinguished on the basis of the surface area lobe and for the blue component −5. The evaluation given above is only a subjective interpretation of the correlation and is intended to introduce arguments for class reduction. By simple summation of the classes, we obtain a set of 12 elements with different combinations. Therefore, it is reasonable to create sets of classes with the smallest number of 12 elements.

Table 2 summarizes the results of the learning process for different network configurations. The input vector was an ordered set of fractal dimensions and the area of red component change. This table presents the results that prove that Kohonen’s neural network with an increase in the number of classes minimizes the learning error to even smaller values. It was observed that for all class sizes, the learning error is below 5%. The error of testing networks above 5% occurs only in structure 3 × 5. The results can be considered promising because the size of the learning set was 70% of the total set. Due to small class variation, the error values range from 1% to 5% for all structures.

The analysis of classes of Kohonen’s artificial neural network algorithm allows us to conclude that the selected method generalizes the acquired knowledge and can be a useful tool in further work on the application of this method in support of dermatological diagnostics.

## 4. Discussion

The characteristics of dermatological lesions presented in the first section allow building basic information in the form of a set of the following features: symmetry, regularity of shape, sharpness of the object edges, brown intensity, red intensity and structure.

Studies carried out in recent years [35,36,37,38,52,53] reported that machine learning classification algorithms distinguish suspicious from non-suspicious skin lesions. These studies are geared towards the development of automated diagnosis systems, but the presence of the anisotropy of the images makes the automated analysis of dermatological images a difficult task [40]. In [41], the images were classified using their morphological structure, color and texture properties and fractals the first time. 

Our research proves that fractal analysis introduces additional information that may be supplementary to the set of input features. Therefore, a preliminary classification of dermatological lesions based on fractal features was conducted.

The classification methods of dermatological lesions described in the previous section will help the physician to make a diagnosis and classify the nevus into the appropriate group. They are based on generally accepted patterns, and each newly created scale leads to greater effectiveness in comparison with the previous one. The scales used by physicians are aimed at characterizing the naevus based on accepted patterns. Most dermatological naevi have an irregular shape, which is even impossible to analyze with a naked eye, and therefore fractal analysis seems to be a suitable tool to measure surface irregularities.

Research results presented in this paper allow formulating the following conclusions. First, the analysis of fractal surface dimension should be supplemented by fractal analysis of the pigmented lesion edge. Additionally, optimization of image parameters, reduction, and supplementation of the feature vector is an element necessary for subsequent stages of classification, both unsupervised and supervised.

Conducted studies and analysis of the obtained results highlight the problem in dermatological diagnostics, which is the qualification of pigmented skin lesions. However, considering the image in terms of morphological features only cannot be considered as a complete analysis in the sense of classification automatics.

The experience throughout many years has allowed dermatologists to develop several methods (scales) that help characterize the dermatological changes under study. The most popular are classical pattern analysis, ABCD scale (sometimes extended to ABCDE), CASH algorithm, Clark and Breslow scale, TNM scale and the seven-point checklist.

Modern dermatoscopic devices, combined with specialized software, provide better opportunities to control and monitor the dermatological features, which, combined with the growing awareness of patients, allow early detection (and thus treatment) of alarming signs of neoplastic changes. Symmetry, texture, border fluidity and color are not a complete objective set for the diagnosis. The identification machine does not have the intuition of an experienced physician and is based only on the knowledge learned from the input vector with a qualitative variable. The fact that the knowledge base needs to be expanded with an increasing number of features is a result of these deliberations.

Since advanced melanoma remains practically incurable, early detection of the lesion is the most important step toward a reduction in mortality. However, evaluation of pigmented skin lesions only based on the ABCDE rule or Seven Point Checklist is extremely subjective and often insufficient. Using fractal parameters in dermatology can be useful for the screening process to provide fully automatic diagnostic systems capable of determining the type of pigmented skin lesion, as it can inform the decision to excise precociously malignant lesions or to avoid unnecessary removal of benign ones. The good diagnostic performance of our method constitutes an important step in the direction of automated diagnosis of pigmented skin lesions in the future.

## Figures and Tables

**Table 1:** Comparison of the winning neurons for three test trials in the Kohonen 8 × 8 network.

Neuron Position	Activation	Dr−01	Dg−01	Db−01	Ds−01	Ar−01	Ag−01	Ab−01	As−01
(1, 5)	3.78	2.20	2.20	2.21	1.12	5.38	5.75	6.00	6.00
(4, 1)	0.10	2.07	2.08	2.12	2.09	0.91	1.04	1.24	1.24
(8, 1)	0.28	2.44	2.27	2.13	2.28	6.19	7.49	5.37	5.37

**Table 2:** Comparison of Kohonen network structures as well as learning and testing error parameters for the five-element learning vector.

Structure	Number of Classes	Number of Eras	Learning Error	Test Error
3 × 4	12	10,000	3.1%	3.9%
2 × 7	14	10,000	2.6%	3.6%
3 × 5	15	10,000	4.2%	5.5%
4 × 4	16	10,000	2.6%	3.6%
2 × 8	16	10,000	2.7%	3.6%
9 × 2	18	10,000	2.3%	3.6%
3 × 6	18	10,000	2.1%	3.7%
4 × 5	20	10,000	1.4%	3.5%
3 × 7	21	10,000	1.8%	3.1%
11 × 2	22	10,000	1.4%	3.4%
4 × 6	24	10,000	1.0%	3.3%

## Data Availability

Data sharing is not applicable. No new data were created or analyzed in this study. Data sharing is not applicable to this article.

## References

[1] Blum A., Hofmann-Wellenhof R., Luedtke H., Ellwanger U., Steins A., Roehm S., Garbe C., Soyer H. (2004). Value of the clinical history for different users of dermoscopy compared with results of digital image analysis. J. Eur. Acad. Dermatol. Venereol..

[2] Capdehourat G., Corez A., Bazzano A., Alonso R., Musé P. (2011). Toward a combined tool to assist dermatologists in melanoma detection from dermoscopic images of pigmented skin lesions. Pattern Recognit. Lett..

[3] Kittler H. (2007). Dermatoscopy: Introduction of a new algorithmic method based on pattern analysis for diagnosis of pigmented skin lesions. Dermatopathol. Pract. Concept..

[4] Placek W. (1996). Wykwity skórne i stany narzucone. Wybrane Pojęcia z Dermatologii—Encyklopedia Badań Medycznych.

[5] Ruiz D., Berenguer V., Soriano A., SáNchez B. (2011). A Decision support system for the diagnosis of melanoma: A comparative approach. Expert Syst. Appl..

[6] Zalewska-Janowska A., Błaszczyk H. (2009). Choroby Skóry.

[7] Miedziński F. (1982). Melanodermie i inne choroby barwnikowe. Dermatologia.

[8] Tschandl P., Rosendahl C., Kittler H. (2018). The HAM10000 dataset, a large collection of multi-source dermatoscopic images of common pigmented skin lesions. Sci. Data.

[9] Brú A., Albertos S., Luis Subiza J., García-Asenjo J.L., Brú I. (2003). The universal dynamics of tumor growth. Biophys. J..

[10] Korotkov K., Garcia R. (2012). Computerized analysis of pigmented skin lesions: A review. Artif. Intell. Med..

[11] Kuczyński K. (2008). Klasyfikacja Obrazów Radiologicznych Na Podstawie Wymiaru Fraktalnego. Sci. Bull. Chełm Sect. Math. Comput. Sci..

[12] Okuboyejo D.A., Olugbara O.O., Odunaike S.A. Automating Skin Disease Diagnosis Using Image Classification. Proceedings of the World Congress on Engineering and Computer Science.

[13] Przystalski K. (2014). Detekcja i Klasyfikacja Barwnikowych Zmian Skóry na Zdjęciach Wielowarstwowych. Ph.D. Thesis.

[14] Johr R.H. (2002). Dermoscopy: Alternative melanocytic algorithms—The ABCD rule of dermatoscopy, menzies scoring method, and 7-point checklist. Clin. Dermatol..

[15] Stolz W., Reimann A., Cognetta A.B. (1994). ABCD rule of dermatoscopy: A new practical method for early recognition of malignant melanoma.

[16] Baish J.W., Jain R.K. (2000). Fractals and cancer. Cancer Res..

[17] Falconer K.J. (2014). Fractal Geometry: Mathematical Foundations and Applications.

[18] Mandelbrot B.B. (1983). The Fractal Geometry of Nature/Revised and Enlarged Edition.

[19] Xu S., Weng Y. (2006). A New approach to estimate fractal dimensions of corrosion images. Pattern Recognit. Lett..

[20] Kunze H., La Torre D., Mendivil F., Vrscay E.R. (2012). Fractal-Based Methods in Analysisi.

[21] Unlu E., Akay B.N., Erdem C. (2014). Comparison of dermatoscopic diagnostic algorithms based on calculation:The abcd rule of dermatoscopy, the seven-point checklist, the three-point checklist and the cash algorithm in dermatoscopic evaluation of melanocytic lesions. J. Dermatol..

[22] Lawrence M.J., Sabra A., Thomas P., Obaid D.R., D’Silva L.A., Morris R.H.K., Hawkins K., Brown M.R., Williams P.R., Davidson S.J. (2015). Fractal dimension: A novel clot microstructure biomarker use in st elevation myocardial infarction patients. Atherosclerosis.

[23] Beckers F., Verheyden B., Couckuyt K., Aubert A.E. (2006). Fractal dimension in health and heart failure. Biomed. Tech. (Berl).

[24] Zheng Y., Keller B.M., Ray S., Wang Y., Conant E.F., Gee J.C., Kontos D. (2015). Parenchymal texture analysis in digital mammography:A fully automated pipeline for breast cancer risk assessment. Med. Phys..

[25] Daye D., Keller B., Conant E.F., Chen J., Schnall M.D., Maidment A.D.A., Kontos D. (2013). Mammographic parenchymal patterns as an imaging marker of endogenous hormonal exposure:A preliminary study in a high-risk population. Acad. Radiol..

[26] Sindeaux R., de Souza Figueiredo P.T., de Melo N.S., Guimarães A.T.B., Lazarte L., Pereira F.B., de Paula A.P., Leite A.F. (2014). Fractal Dimension and mandibular cortical width in normal and osteoporotic men and women. Maturitas.

[27] Use of Fractal Analysis in Dental Images for Osteoporosis Detection: A Systematic Review and Meta-Analysis. https://link.springer.com/article/10.1007/s00198-021-05852-3.

[28] Huang C.C., Chen J.C., Chang Y.C., Jeng J.H., Chen C.M. (2013). A fractal dimensional approach to successful evaluation of apical healing. Int. Endod. J..

[29] Carpinteri A., Lacidogna G., Niccolini G. (2009). Fractal analysis of damage detected in concrete structural elements under loading. Chaos Solitons Fractals.

[30] Shu Z.R., Chan P.W., Li Q.S., He Y.C., Yan B.W. (2020). Quantitative assessment of offshore wind speed variability using fractal analysis. Wind Struct..

[31] Kabaldin Y., Anosov M., Shatagin D. (2020). Evaluation of the mechanism of the destruction of metals based on approaches of artificial intelligence and fractal analysis. IOP Conf. Ser. Mater. Sci. Eng..

[32] Nanoscale Morphology and Fractal Analysis of TiO_2_ Coatings on ITO Substrate by Electrodeposition—Amâncio—2021—Journal of Microscopy Wiley Online Library. https://onlinelibrary.wiley.com/doi/abs/10.1111/jmi.12990.

[33] Sadeghi B. (2021). Concentration-Concentration Fractal Modelling: A novel insight for correlation between variables in response to changes in the underlying controlling geological-geochemical processes. Ore Geol. Rev..

[34] Category-Based Fractal Modelling: A Novel Model to Integrate the Geology into the Data for More Effective Processing and Interpretation—ScienceDirect. https://www.sciencedirect.com/science/article/abs/pii/S0375674221000613.

[35] Sapota W., Szczepanik P., Stach S., Wróbel Z. (2020). Fractal and multifractal analyses of the porosity degree of ceramics used in biomedicine. Adv. Sci. Eng. Med..

[36] Kiselev V.M., Golovanova O.A., Fedoseev V.B. (2021). The study of modified hydroxylapatite samples using the fractal theory. Crystallogr. Rep..

[37] Analysis of Complex Modal Instability of a Minimal Friction Self-Excited Vibration System from Multiscale Fractal Surface Topography—ScienceDirect. https://www.sciencedirect.com/science/article/abs/pii/S0997753821000206.

[38] Arumugam S., Manoharan S., Palaniswamy S.K., Kumar S. (2021). Design and performance analysis of a compact quad-element UWB MIMO antenna for automotive communications. Electronics.

[39] Sarkar N., Chaudhuri B.B. (1992). An efficient approach to estimate fractal dimension of textural images. Pattern Recognit..

[40] Bisoi A.K., Mishra J. (2001). On calculation of fractal dimension of images. Pattern Recognit. Lett..

[41] Oczeretko E., Rogowski F. (1992). Wymiar fraktalny i jego zastosowanie w biologii i medycynie. Probl. Med. Nukl..

[42] Omiotek Z. (2012). Zastosowania Wymiaru Fraktalnego do analizy konturu obiektów. Inform. Autom. Pomiary Gospod. Ochr. Sr..

[43] Allain C., Cloitre M. (1991). Characterizing the Lacunarity of random and deterministic fractal sets. Phys. Rev. A.

[44] Filho M.N.B., Sobreira F. Accuracy of Lacunarity Algorithms in Texture Classification of High Spatial Resolution Images from Urban Areas. https://www.isprs.org/proceedings/XXXVII/congress/3b_pdf/80.pdf.

[45] Gefen Y., Meir Y., Mandelbrot B.B., Aharony A. (1983). Geometric implementation of hypercubic lattices with noninteger dimensionality by use of low lacunarity fractal lattices. Phys. Rev. Lett..

[46] Khurshid K., Siddiqi I., Faure C., Vincent N. Comparison of Niblack Inspired Binarization Methods for Ancient Documents. Proceedings of the 16th Document Recognition and Retrieval Conference, Part of the IS&T-SPIE Electronic Imaging Symposium.

[47] Sauvola J., Pietikäinen M. (2000). Adaptive Document Image Binarization. Pattern Recognit..

[48] Singh T.R., Roy S., Singh O.I., Sinam T., Singh K. (2012). A new local adaptive thresholding technique in binarization. arXiv.

[49] Chen W.-T., Wen C.-H., Yang C.-W. (1994). A fast two-dimensional entropic thresholding algorithm. Pattern Recognit..

[50] Gupta M.R., Jacobson N.P., Garcia E.K. (2007). OCR binarization and image pre-processing for searching historical documents. Pattern Recognit..

[51] Niblack W. An Introduction to Digital Image Processing.

[52] Chaurasia V., Pal S. (2020). Machine Learning algorithms using binary classification and multi model ensemble techniques for skin diseases prediction. Int. J. Biomed. Eng. Technol..

[53] Hameed N., Shabut A.M., Ghosh M.K., Hossain M.A. (2020). Multi-class multi-level classification algorithm for skin lesions classification using machine learning techniques. Expert Syst. Appl..

